# Evaluation of Glycosyl-Hydrolases, Phosphatases, Esterases and Proteases as Potential Biomarker for NaCl-Stress Tolerance in *Solanum lycopersicum* L. Varieties

**DOI:** 10.3390/molecules24132488

**Published:** 2019-07-07

**Authors:** Juan José Reyes-Pérez, Francisco Higinio Ruiz-Espinoza, Luis Guillermo Hernández-Montiel, Barbara de Lucía, Giuseppe Cristiano, Bernardo Murillo-Amador

**Affiliations:** 1 Facultad de Ciencias Agrarias, Universidad Técnica Estatal de Quevedo, Quevedo, Los Ríos 120501, Ecuador; 2 Departamento Académico de Agronomía, Universidad Autónoma de Baja California Sur, La Paz, Baja California Sur 23080, Mexico; 3 Programa de Agricultura en Zonas Áridas, Centro de Investigaciones Biológicas del Noroeste, S.C. La Paz, Baja California Sur 23096, Mexico; 4 Dipartimento di Scienze agro-ambientali e territoriali, University of Bari Aldo Moro, Bari 70121, Italy

**Keywords:** salinity-stress tolerance, biochemical indicators, proline, peroxidase, semi-quantitative enzymes, tomato

## Abstract

Salinity stress limited the production in over 30% of irrigated crops and 7% of dryland agriculture worldwide. The objective was to evaluate the effects of NaCl-stress on the enzymatic activity in tomato. Two experiments were carried out in germination and early vegetative growth stages. The activity of proline and peroxidase of eight varieties (Missouri, Yaqui, Vita, Feroz, Rio Grande, Tropic, Ace, and Floradade) submitted to NaCl concentrations (0, 50, 100, 150 and 200 mM de NaCl) and the semi-quantitative activity of 19 enzymes APY ZYM^®^ were measured under a completely randomized design with four replications. Data were analyzed using univariate-multivariate analysis of variance, Tukey’s HSD (*p* = 0.05), canonical discriminant and cluster analysis. The results showed significant differences between varieties and NaCl in proline content. Proline increased as the NaCl concentration increased. Peroxidase did no show significant differences. Eight enzymes were included within the model to properly classify the varieties and NaCl. In shoots, varieties and NaCl showed that enzymatic activity was higher in the order of alkaline-phosphatase > leucine arylamidase > acid phosphatase > naphthol-AS-BI-phosphohydrolase > n-acetyl-β-glucosaminidase > β-galactosidase, while in roots was higher in the order of alkaline-phosphatase > naphthol-AS-BI-phosphohydrolase > acid phosphatase > n-acetyl-β-glucosaminidase. Acid and alkali phosphatase, lipase, esterase, β-galactosidase, and trypsin can be a potential biomarker for NaCl-stress tolerance in tomato.

## 1. Introduction

The humanity uses about half of the fresh water freely accessible in the planet to maintain an increasing world population. One of the productive activities that competes with industrial and domestic uses of fresh water is agriculture. On a day to day basis, the high quality fresh water is limited and is a costly resource. Besides the competition for accessible fresh water, which represents 1% of the Earth’s fresh water, is the continuing and permanent spread of salinization [1]. Among all abiotic stresses, salinity is modifying soil and fresh water, mainly in semiarid and arid areas and from all abiotic stresses, salinity is a main contributor in reducing the crop productivity [2]. The Food and Agriculture Organization of the United Nations (FAO) [3] cited that over 6% of the world’s land is disturbed by salinity covering around 400 Mha of the world’s land area. Salinization is quickly expanding on a worldwide scale and now affects more than 10% of arable land, which results in a failure rate of the normal yields of main crops of more than 50% [4].

Tomato is an extensively dispersed vegetable crop which is consumed cooked or fresh and also processed as sauces, juices, paste and others. Tomato as a crop has a good adaptation to different climatological conditions since it can be cultivated from tropical areas to within a few degrees of the Arctic Circle [5,6]. This species has the highest economic importance in the world; however, salinity stress causes a reduction in the quantity and quality of tomato production. The salinity of soil and water is amongst the most significant abiotic stresses and this environmental stress reduces agricultural production worldwide [7]. Consequently, it is imperative to comprehend how plants react and adapt to such types of stresses. Some studies reported that tomato is sensitive to moderately sensitive to salt stress [8,9,10]. Other studies reported that tomato is to be moderately tolerant with 50% yield losses at 7.5–8 dS m^−1^ [11,12,13].

Since plants have a sedentary mode of life, they cannot escape to different abiotic environments as animals have done. In this sense, plants must develop different adaptive strategies in response to stresses such as salinity, drought, heat, cold, and nutrient imbalances (including mineral toxicities and deficiencies) that affect plant growth and productivity [14]. Some of these strategies involve changes in physiological, morphological and biochemical responses [15]. The biochemical responses of plants have been associated to the accumulation of organic components such as sugars, amino acids, quaternary ammonium compounds with osmotic activity [16]. Other osmotically active solutes include polyols, betaine, trehalsose, ectoine, proline, and others [17]. Proline has been extensively investigated because it is one of the most frequent osmolytes for osmoprotection. Similarly, plants under salt stress use a defense strategy against the reactive oxygen, which is a product of hyperosmotic oxygen species that naturally induces the activity of specific antioxidative enzymes. Non-enzymatic antioxidants include carotenoids, tocopherols, ascorbate, glutathione, and phenolic compounds, while enzymatic ones include glutathione transferase, superoxide dismutase, ascorbate peroxidase, catalase, glutathione, peroxidase, and an increase of lipid peroxidation products which include a membrane catabolism [18,19,20]. Another biochemical response of the plants involves the modulation of different enzymes [21,22,23].

These enzymes include glycosyl-hydrolases, phosphatases, esterases and proteases. Some studies have associated some of these enzymes with biotic and abiotic stresses such as drought and salinity. Those enzymes related to salinity stress are esterase, which increases as salinity increased [24,25,26,27,28,29], acid and alkali phosphatase are extensively allocated in plants [30] and are related to salinity stress [18,19,20,30,31,32,33,34,35,36,37,38].

Despite all the data collected, there is a scarcity of information on the precise responses of tomato under NaCl-stress and the induction of isozymes, mainly the changes in various enzymes in tomato varieties with differential responses to NaCl-stress in two growth stages. In this study, we studied the activity of glycosyl-hydrolases, phosphatases, esterases and proteases using tomato as a plant model and we assumed that inter-varietal differences in NaCl-stress could be revealed in the activities of these enzymes, and perhaps that these can be used as potential biomarkers of NaCl-stress for salt tolerance in plant breeding programs; since some varieties used in this study such as Missouri, Rio Grande and Ace, were reported previously with differential responses to NaCl-stress [39,40,41,42,43,44].

Tomato plants can synthesize proline and other osmotically active compounds/protectors and other enzymes. Therefore, these crops could provide a useful model for analyzing the differential effect of NaCl-stress and the synthesis of some osmotic compounds such as proline, peroxidas,e and other enzymes not directly related to antioxidant action in tomato varieties. The objective of the present study was to determine the effects of NaCl-stress on the quantitative activity of proline and peroxidase and semi-quantitative activity of 19 enzymes of the APY ZYM^®^ system in shoots and roots of seedlings of tomato varieties.

## 2. Results

### 2.1. First Experiment

Analysis of MANOVA showed significant differences between tomato varieties (Wilks = 0.181; F = 22.95; *p* ≤ 0.0001), NaCl concentrations (Wilks = 0.398; F = 17.39; *p* ≤ 0.0001) and the interaction of varieties × NaCl (Wilks = 0.235; F = 4.50; *p* ≤ 0.0001). The relationship of Wilks is significant (*p* ≤ 0.01); this confirms that there are variations between the factors in study in the variables that were evaluated, and reinforces the probability that the variations detected in the univariate analysis (ANOVA) were achieved on the variables, are accurate differences and not untrue positives or variations that only occur randomly [45].

#### 2.1.1. Proline Content

Proline displayed significant differences between varieties (*p* ≤ 0.0001), NaCl (*p* ≤ 0.0001) and the interaction of varieties × NaCl (*p* ≤ 0.0001). Proline content increased as NaCl concentrations increased (*r* = 0.38; *R*^2^ = 0.151; *p* = 0.0001, N = 160) showing the highest values at 200 mM NaCl and the lowest at 0 mM NaCl (Table 1). The interaction varieties × NaCl showed that Floradade followed by Feroz displayed the highest proline value at 0 mM NaCl; in 50 and 100 mM NaCl, also Floradade showed highest values, followed by Vita at 50 mM and Yaqui, Feroz, Vita and Rio Grande at 100 mM. Vita exhibited highest values of proline at 150 and 200 mM followed by Rio Grande, Tropic, Floradade, Feroz and Yaqui at 150 and Missouri, Yaqui and Rio Grande at 200 mM NaCl (Table 1).

#### 2.1.2. Peroxidase Activity

Peroxidase did no show significant differences among varieties (*p* ≥ 0.16), NaCl (*p* ≥ 0.70) and the interaction of varieties × NaCl (*p* ≥ 0.77). The analysis of the interaction of varieties × NaCl displayed numerical differences of varieties in the NaCl concentrations. Yaqui showed higher peroxidase activity at 0, 50 and 200 mM; Ace and Floradade showed higher activity at 100; Floradade and Tropic revealed higher activity at 150 mM. Missouri showed lower activity at 0 and 150 mM, Feroz at 50 mM and Tropic at 100 and 200 mM NaCl. The averages values of peroxidase activity with regard to the interaction varieties × NaCl revealed no decreasing or increasing trend of peroxidase activity as the NaCl concentration increased or decreased (Table 1).

### 2.2. Second Experiment

#### 2.2.1. Semi-Quantitative Enzymatic Activity

##### Canonical Discriminant Analysis

**Figure 1:**
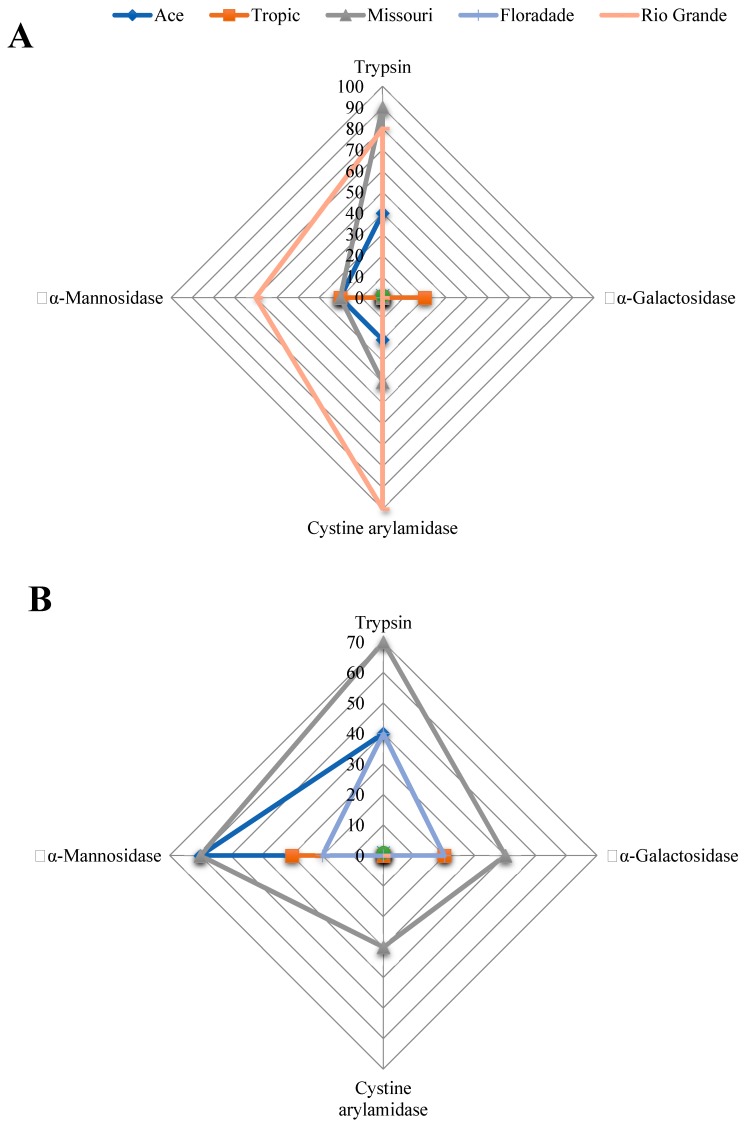

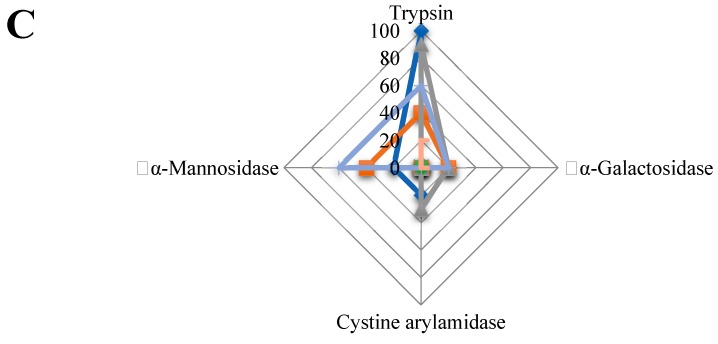
Mean enzymatic activity by the API ZYM from shoots of seedlings from five tomato varieties subjected to (**A**) 0 mM NaCl; (**B**) 50 mM NaCl and (**C**) 100 mM NaCl. Enzymatic activities shown are those designated as the greatest explicatory activities in canonical discriminant analysis revealing significant differences (*p* < 0.05) between the tomato varieties.

A stepwise canonical discriminant analysis was carried out for choosing the maximum explanatory enzymatic activity of shoots and roots according to the tomato varieties subjected to three NaCl concentrations. Consequently, out of the results of shoots plantlets for the 19 enzymatic activities, eight were included within the model to appropriately categorize the five tomato varieties in their respective NaCl concentration and from those, four (trypsin, cystine arylamidase, α-galactosidase and α-mannosidase) had the greatest influence on the discriminant function (Wilks Lambda = 0.108; F_32,178_ = 4.62; *p* ≤ 0.0001) and showed significant differences (*p* ≤ 0.0001) between tomato varieties. In 0 mM NaCl, Rio Grande showed the highest values of enzymatic activity followed by Missouri, while the lowest was showed by Floradade with Ace and Tropic with intermediate values (Figure 1A). In 50 mM NaCl, Missouri exhibited the greatest values of enzymatic activity followed by Ace and Floradade. The lowest values of enzymatic activity were showed by Rio Grande and Tropic with intermediate values (Figure 1B). In 100 mM NaCl, Ace, Missouri and Floradade showed the highest values of enzymatic activity, Tropic showed intermediate values while Rio Grande showed the lowest values (Figure 1C). The carefully chosen discriminant model accurately classified 53.33% of enzyme according to the tomato varieties, with 100% of enzymes from Missouri being adequately classified, followed by Floradade (83.33%), Tropic (50%), Rio Grande (25%) and Ace (8.33%).

**Figure 2:**
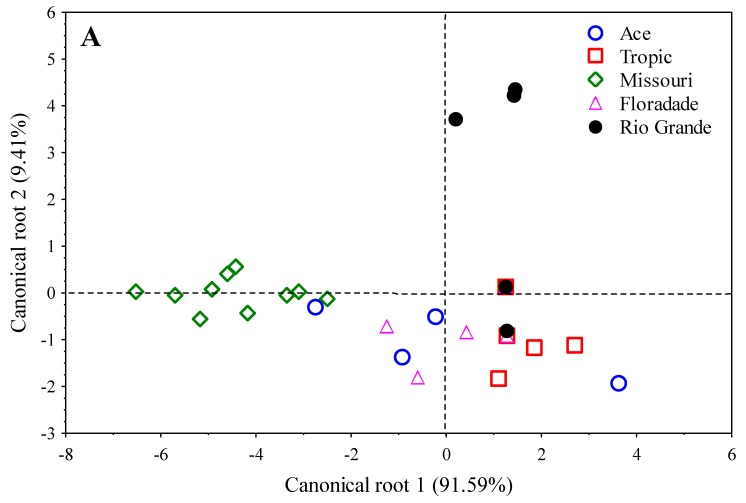

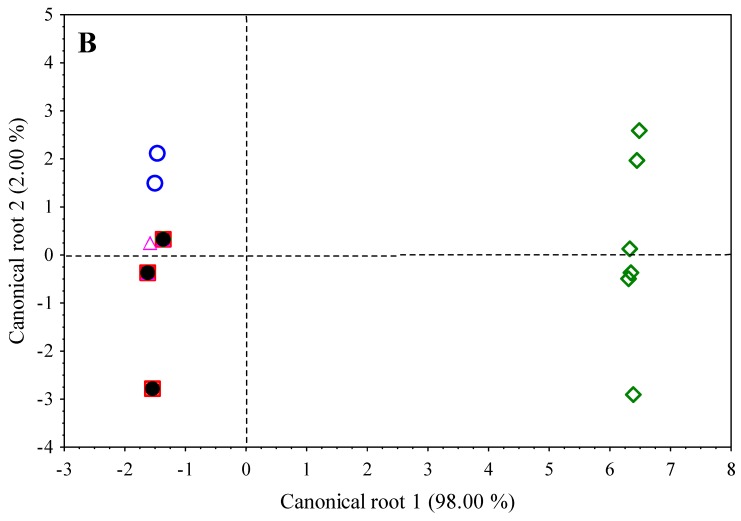
Classification of five tomato varieties germinated under three NaCl concentrations (0, 50 and 100 mM NaCl) established on the first and second canonical meanings (roots), from the canonical discriminant analysis for mean enzymatic activities as determined by API ZYM^®^ in (**A**) shoots and (**B**) roots.

Selected enzymes that were included in the discriminant model for tomato varieties subjected to three NaCl concentrations were then applied in a canonical analysis. In this analysis, two canonical functions (roots) were extracted. In all arrangements, the first canonical function accounted for 91.59% of the variation, while second canonical functions accounted for the remaining 9.41% of the variation (Figure 2A). Figure 2 is bi-plot shows indicating the enzymes projected on the plane of one and two canonical roots. Varieties were grouped according to the API ZYM^®^ enzymes. The differentiation among varieties was relatively evident. Missouri was located in the opposite side of the other varieties (intermediate left-side), Rio Grande was found in the upper-right side, in contrast to the other varieties, while the rest of varieties were positioned in the low-center of the plane, with some of them sharing data in left or right sides, while Tropic was situated on the lower-right side. In general terms, the graphical representation of the varieties showed overlap, but it was not possible to discriminate among varieties with the desired accuracy. The degree to which these five varieties were separated as measured by the Mahalanobis distance between centroid values of the different varieties, no revealed significant differences (*p* = 0.20) for all pairwise distances between them.

The results of seedling roots for the 19 enzymatic activities showed that three were incorporated inside the model to appropriately arrange the five varieties in their respective NaCl concentrations and from those, one (cystine arylamidase) had the greatest influence on the discriminant function (Wilks Lambda = 0.085; F_12,140_ = 17.98; *p* ≤ 0.0001) and showed significant differences (*p* ≤ 0.0001) among varieties. However, in the three NaCl concentrations, only Missouri showed enzymatic activity with cystine arylamidase, while the rest of the varieties did not show activity. The sensibly chosen discriminant model correctly classified 48.33% of enzymes according to the varieties, with 91.66% of enzymes from Missouri being adequately classified, followed by Rio Grande (83.33%), Tropic (50%), Ace (16.66%) and Floradade (0%). Selected enzymes that were integrated in the discriminant model for varieties subjected to three NaCl concentrations were then used in a canonical analysis. In this analysis, two canonical functions (roots) were extracted. In all arrangements, the first canonical function accounted for 98.0% of the variation, while second canonical functions accounted for the residual 2.0% of the variation (Figure 2B). Figure 2 is a bi-plot and shows the enzymes projected on the plane of canonical roots. Varieties were grouped according to the API ZYM^®^ enzymes. The differentiation among varieties was marked. Missouri was in the opposite side to the rest of the varieties (in the upper and lower right-side. The graphical representation of the varieties showed overlap, then, was not possible to discriminate among varieties with the wanted exactness. The degree to which the varieties were distinguished as determined by the Mahalanobis distance among centroid values of the varieties, no specified significant differences (*p* = 0.20) for all pairwise distances between them.

##### Cluster Analysis

**Figure 3:**
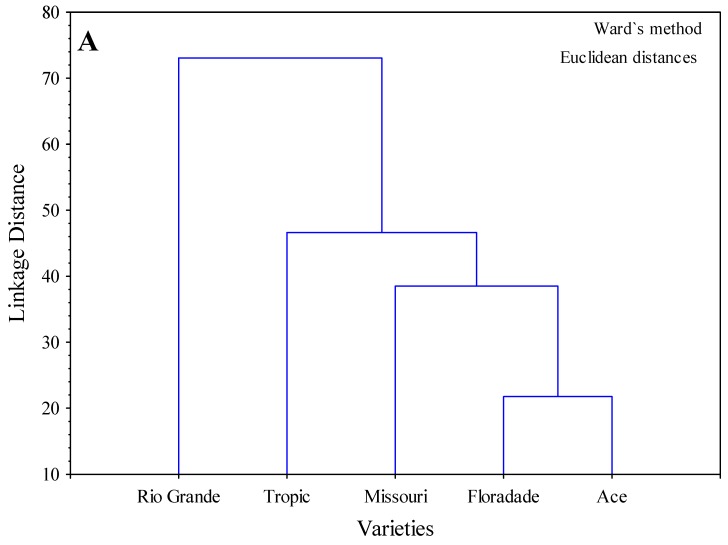

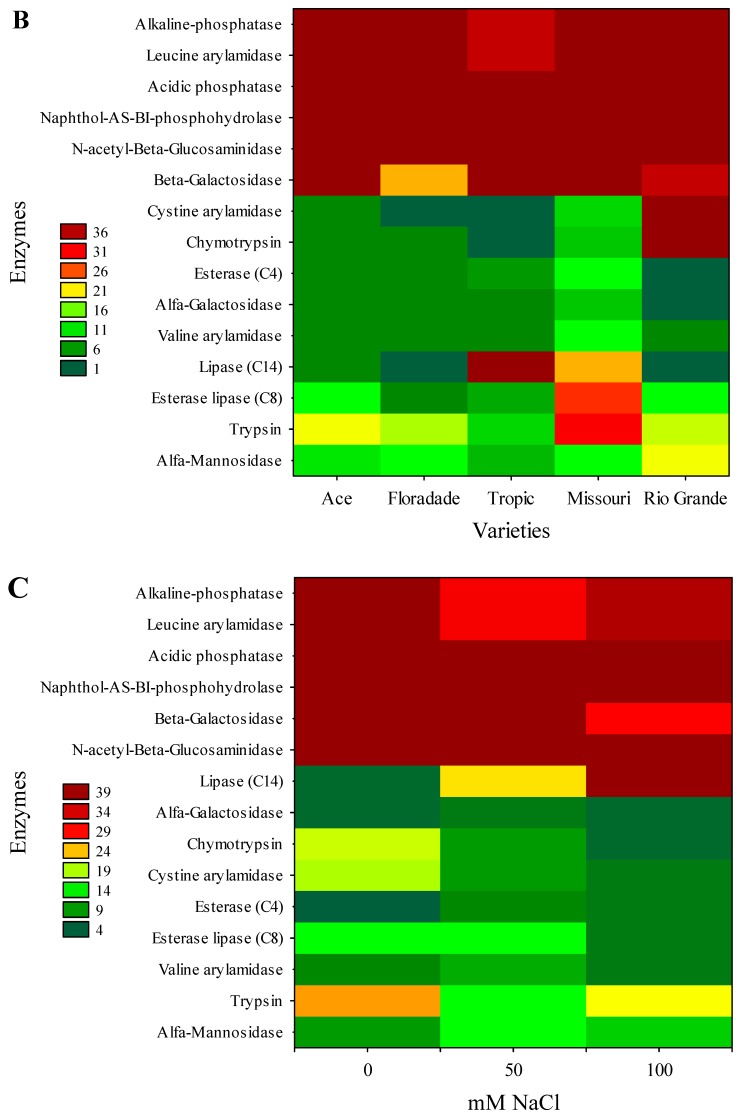
Cluster analysis of merged data from 19 enzymatic activities as quantified by the API ZYM^®^ in five tomato varieties (SHOOTS) subjected to three NaCl concentrations. The dendrogram shows (**A**) the grouping of varieties using the similarity matrix (Ward algorithm) created from the strength of the different activities (from 0 to 40 nmol) applying the Euclidean distance coefficient. The enzymatic activity using the two-ways joining clustering method (**B**) among varieties and (**C**) among NaCl concentrations. Strength of the color in the figures is related to enzyme activity in agreement with the two-ways joining clustering method.

The cluster developed with the pooled analysis of enzymatic activities with the API ZYM^®^ of shoots of seedlings of five varieties subjected to three NaCl concentrations revealed a structure in the grouping of data used to produce it. The dendrogram shows a first group formed by Ace and Floradade, a second group formed by Missouri, a third group formed by Tropic and a fourth group formed by Rio Grande (Figure 3A). The two-ways joining clustering method among varieties showed that enzymatic activity was higher in most varieties in the order of alkaline-phosphatase > leucine arylamidase > acid phosphatase > naphthol-AS-BI-phosphohydrolase > n-acetyl-β-glucosaminidase > β-galactosidase. The enzymatic activity of cystine arylamidase and chymotrypsin was high in Rio Grande and lipase (C14) in Tropic. Other enzymes showed lower values in all varieties (Figure 3B). The analysis of the two-ways joining clustering method among NaCl concentrations showed that enzymatic activity was higher at the three NaCl concentrations in the order of alkaline-phosphatase > leucine arylamidase > acid phosphatase > naphthol-AS-BI-phosphohydrolase > β-galactosidase > n-acetyl-β-glucosaminidase. Out of these enzymes, only lipase (C14) exhibited a tendency to increase as NaCl concentration increased (Figure 3C).

**Figure 4:**
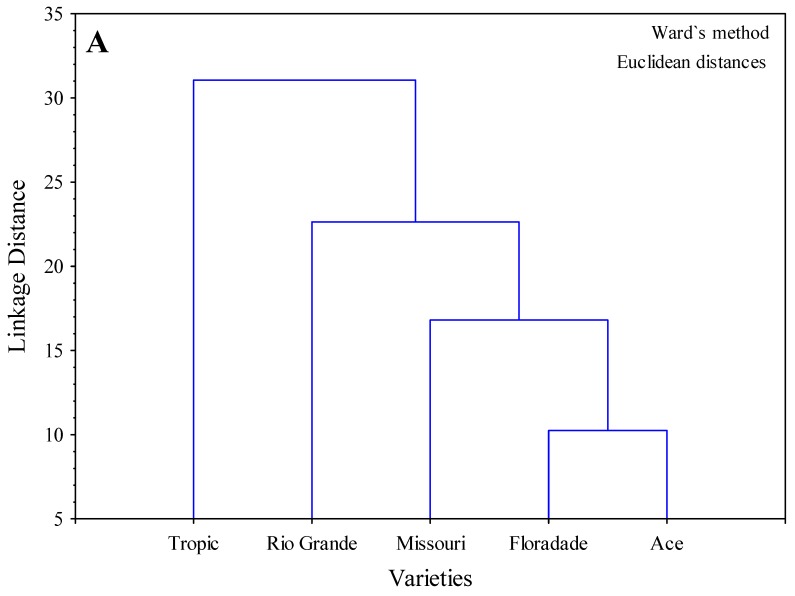

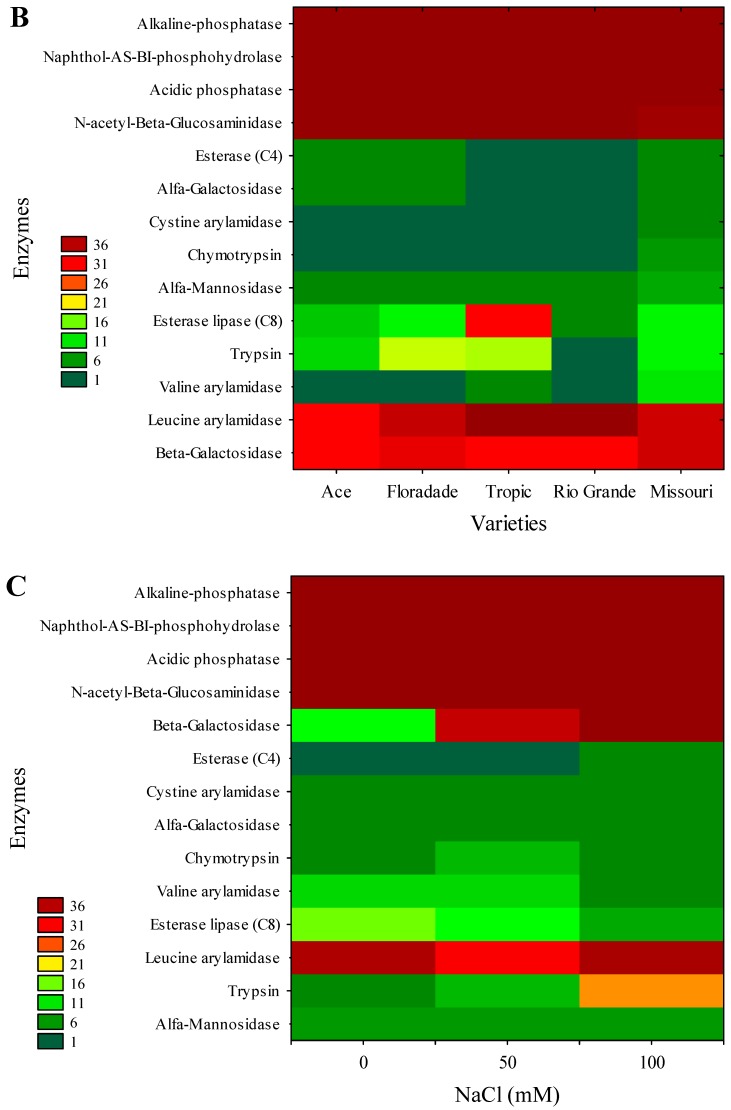
Cluster analysis of merged data from 19 enzymatic activities as determined by the API ZYM^®^ in five tomato varieties (ROOTS) subjected to three NaCl concentrations. The dendrogram shows (**A**) the grouping of varieties using the similarity matrix (Ward algorithm) created from the strength of the different activities (from 0 to 40 nmol) applying the Euclidean distance coefficient. The enzymatic activity using the two-ways joining clustering method (**B**) among varieties and (**C**) among NaCl concentrations. Strength of color in the figures is related to the enzyme activity in agreement with the two-ways joining clustering method.

The cluster completed with the pooled evaluation of enzymatic activities with the API ZYM^®^ of the roots and plantlets of five varieties subjected to three NaCl concentrations, which revealed a specific structure in the grouping of data used to create it. The dendrogram showed a first group formed by Ace and Floradade, a second group formed by Missouri, a third group formed by Rio Grande and a fourth group formed by Tropic (Figure 4A). The analysis of two-ways joining clustering method between varieties revealed that major enzymatic activity was in the order of alkaline-phosphatase > naphthol-AS-BI-phosphohydrolase > acid phosphatase > n-acetyl-β-glucosaminidase. The enzymatic activity of leucine arylamidase showed relatively higher values in all varieties, particularly in Tropic and Rio Grande. Also, β-galactosidase showed moderate values among varieties. The rest of enzymes showed lower values in all varieties (Figure 3B). The analysis of the two-ways joining clustering method among NaCl concentrations showed that enzymatic activity was in the order of alkaline-phosphatase > naphthol-AS-BI-phosphohydrolase > acid phosphatase > n-acetyl-β-glucosaminidase. Also, leucine arylamidase showed higher values in all NaCl concentrations. Some enzymes such as β-Galactosidase, trypsin and esterase showed a trend to increase as the NaCl concentration increased (Figure 4C).

## 3. Discussion

In the present study, the NaCl-stress effect in tomato varieties was investigated by measuring the proline content, peroxidase activity and semi quantitative enzymatic activities estimated by the API ZYM^®^, in order to verify the roles of organic osmolytes and enzymatic activities and to determine differences among varieties subjected to NaCl concentrations. In this study, proline content differed among varieties. From 1979 until the present, various studies have examined tomato under NaCl stress to demonstrate that proline content increased as the NaCl concentration increased. Some recent studies reported similar results to those obtained in the present study, i.e., Nouck et al. [46] evaluating cultivars of *Lycopersicum esculentum* with differences in salt-tolerance found that amino acids, specifically proline, increased in salt-tolerant cultivar, suggesting that proline can be used as a biochemical marker of initial selection and osmotic amendment capacity for salt-tolerant plants.

Proline accrual in NaCl-stressed plants might be a result of the low activity of the oxidizing enzymes [47]. Also, it has been indicated that leaves accumulate more proline in order to preserve the chlorophyll level, and cell turgor to retain photosynthetic activity under salt stress [48]. This is in agreement with the results of the present study, indicating that proline content was highest in the leaves of tomato plants subjected to NaCl-stress. Tissue accumulation of proline under NaCl stress could result largely from *de-novo* biosynthesis, since proline increased as NaCl concentrations increased [49]. However, other studies have suggested that a confirmed association among an increase of proline in plants under stress is a product of, and not an adaptive reaction to stress. Nevertheless, according to Bolarin et al. [50] proline increase in leaves and, mainly, in roots is considered as a salt sensitive characteristic in tomato that can be applied to selected plants with different levels of salt tolerance.

Regarding proline and their relationship with NaCl concentration, the information reported here is not totally new; the results confirm that at least in tomato, proline increased as NaCl concentrations increased and that proline content showed differences among varieties. Similar results have been reported showing that tomato plants subjected to NaCl generate osmotically active organic constituents that can lessen the effect of salinity caused by NaCl, which was reported some years ago by Storey and Wyn Jones [51], where proline content was 10-fold greater in shoots and 18-fold greater in roots of tomato plants grown at 100 mM NaCl than in plants grown without salinity. This response is also maintained in tomato wild species such as *Lycopersicon pennellii* with greater faculty of osmotic adjustment even more than *Lycopersicon esculentum* [52]. More recent studies using tomato as the model plant have shown that proline content increased as NaCl stress increased [53,54,55,56,57,58,59,60,61].

As previously mentioned, peroxidase do not show significant differences among varieties, NaCl concentrations and the interaction varieties × NaCl; however, Yaqui showed highest peroxidase activity at 50 and 200 mM NaCl and Floradade at 100 and 150 mM NaCl. Similar effects were reported in tomato under salinity effects by Halo et al. [62], where the activities of catalase, peroxidase and polyphenol oxidase were either lower or non-significant as related to control. This study showed that peroxidase activity no revealed a trend to decrease or increase as NaCl concentration increased or decreased. Similar responses of peroxidase activity on tomato varieties under NaCl-stress were previously reported by Kanokwan et al. [63], who described that SOD, CAT and GPx exhibited increase and decrease trends that closely followed the same pattern in salinity reactions but without the same activity levels. Nevertheless, when salicylic acid was applied to mitigate salt-stress in tomato, it caused a tenfold increase in ascorbate peroxidase (APX) activities of young leaves and significant increases in APX and glutathione reductase (GR) activities of the roots [64].

Other studies showed that wild tomato species was more salt tolerant than cultivated tomato, while inclusive wild species showed better protection to salt-stress inducing oxidative stress, which was associated with increased activities of APX (ascorbate peroxidase), SOD and POD (guiacol peroxidase) [65,66,67,68]. Some reports of antioxidant responses to salinity stress such as peroxidase activity are from 40 years ago [69]. Significant studies concluded that water deficit as a result of osmotic effects occurs when plants are submitted to salt stress, provoking oxidative stress derived from the development of reactive oxygen species for example hydroxyl, superoxide, and peroxyl radicals; however, the reactive oxygen species that are by-products of ionic and hyperosmotic stresses affect membrane dysfunction and provoke cell collapse [70].

The antioxidative enzymes such as superoxide dismutase, peroxidase, glutathione reductase, catalase and others, are derived from the plants as defend system versus the reactive oxygen species which scavenge reactive oxygen species. This mechanism is detected in tomato under NaCl-stress; despite the high value of the tomato to humans, little is known about the changes and ways of NaCl tolerance in currently consumed commercial tomato varieties, especially since the studies under field conditions are scarce. Nevertheless, recent reports point out that Rio Grande is a salinity-tolerant cultivar [57,71]. This variety was used in the present study and showed high proline content at 100, 150 and 200 mM NaCl as well as relative high peroxidase activity at 200 mM NaCl; however, this variety was the least NaCl tolerant when physiological variables such as stomatal conductance, water potential, chlorophyll, transpiration, and other variables were measured [72].

This result shows that an obvious correlation cannot continually be found among the physiological response of genotypes under NaCl-stress and the induction of antioxidant enzymes, i.e., the genotype not necessarily will show NaCl-tolerance in physiological, morphometric and biochemical variables at the same time. This differential response can be attributed to the mechanical ability of each genotype to tolerate NaCl-stress or because of the effect of NaCl-stress that may cause some changes in gene expression. In other species such as *Brassica*, peroxidase activity in response to salinity is not a consistent criterion to select for tolerance [69,73].

Arbona et al. [74] reported that the adverse effect on the growth of citrus rootstock was principally a result of a cellular intoxication by Cl and was not due to salt-induced oxidative stress. Nevertheless, the majority of studies reported that peroxidase activity increased as a response of salinity stress in some species such as *Alhagi maurorumv* [29], rice [75], cucumber [76], sorghum [77], cotton [78,79], and cowpea [80]. Generally, the studies reported that enzyme activity usually increases under stresses caused by salinity, drought, high or low temperature, ozone, and toxic compounds such as pesticides and heavy metals. However, there are in the literature some conflicting reports about the relationship of enzyme activity and salinity stress tolerance; but the results reported suggest that the enzymatic activity can be used a biomarker for NaCl-stress tolerance. The majority of studies of enzymatic activity have been developed using seeds, seedlings or plants in the early growth stage; nevertheless, from the productive and economic perspective, the most important variables are the yield and the quality of the fruits. As such, field studies in the productive phenological stage of cultivated species such as tomato under NaCl-stress are required.

The use of the API ZYM^®^ system to determine semi-quantitative enzymatic activity in tomato and their relationship with NaCl-stress tolerance was effective to demonstrate that other enzymes that are not widely studied in plants show a relationship with NaCl-stress tolerance. In this context, from the 19 enzymatic activities detected by API ZYM^®^, eight enzymes were involved in the model to appropriately order the five varieties in their corresponding NaCl concentration. In shoots four enzymes (trypsin, cystine arylamidase, α-galactosidase and α-mannosidase) and in roots only one enzyme (cystine arylamidase) had the highest contribution to the discriminant function and showed significant differences between tomato varieties.

In this study when the analysis of enzyme activity in shoots was carried out by varieties and by NaCl, the results exhibited that enzymatic activity was higher in tomato varieties in the order of alkaline-phosphatase > leucine arylamidase > acid phosphatase > naphthol-AS-BI-phosphohydrolase > n-acetyl-β-glucosaminidase > β-galactosidase, while in NaCl concentrations the order was alkaline-phosphatase > leucine arylamidase > acid phosphatase > naphthol-AS-BI-phosphohydrolase > β-galactosidase > n-acetyl-β-glucosaminidase. In roots, the enzymatic activity was higher in varieties and NaCl concentrations in the order of alkaline-phosphatase > naphthol-AS-BI-phosphohydrolase > acid phosphatase > n-acetyl-β-glucosaminidase. Phosphatases activity is broadly dispersed in plants and the activity differ among plant species that have been studied [32]. The increase of phosphatases activity as response to NaCl-stress is a new finding in tomato, but not in other plants since acid and alkali phosphatase activity is known to promote tolerance under water and salt stress because it maintains a specific quantity of inorganic phosphate [32]. The acid phosphatase activity trend increased in roots of one variety and decreased in other, while in shoots it showed no difference with the control among lettuce varieties under salinity stress [39].

In this study, both acid and alkali phosphatase increased in shoots and roots as the NaCl concentration increased, therefore, the phosphatase activity of tomato seedlings can be a potential biomarker for NaCl-stress tolerance in this species. This response is because of seedlings under NaCl-stress reduce their growth and the transfer of phosphate is diminished, thus contributing to the stimulation of the cellular phosphatases that liberate soluble phosphate from its insoluble compounds inside or outside of the cells, thereby regulating osmotic adjustment by a free phosphate uptake mechanism. Previous studies reported that NaCl-stress enhance acid and alkali phosphatase activity in canola seeds [38], *Medicago sativa* [23], pearl millet seeds [81], sorghum [82] and cowpea [83].

This study showed that lipase (C14) activity in shoots, esterase, β-galactosidase and trypsin in roots of tomato seedlings showed a trend to increase as NaCl concentration increased. The increase in esterase activity might be associated to NaCl tolerance of the varieties reached through ion accumulation; however, the mineral analysis in the tissues needs to be analyzed. Similar results were reported by Dombrowski [84] when Serine PIs (II) were detected in NaCl-stressed tomato, and Dombrowski proposed that PIs are also implicated in abiotic stress. The increase of trypsin PI activity has a positive effect because it helps to block trypsin proteases that are liable for degradation of specific proteins. Also, chymotrypsin inhibitor was reported in rice under dehydration and abscisic acid treatments [85]. The reduction of chymotrypsin protease activity also has been related with the tolerance to abiotic stress in Arabidopsis [86]. All of these studies have demonstrated that the action of these enzymes occurs in the amino acid level.

The relationship of salinity and esterase activity has been studied in higher plants regardless of their tolerance to salinity stress [24,25,28]. In shoots and roots of maize plants under 150 mM NaCl, Mohamed [87] reported the increase of esterase isozyme and this activity differed between distinct physiological stages of higher plants. In legumes such as peanut [25] and cowpea [80], it was found that salinity increases esterase activity at concentrations of NaCl (25, 100, 150 or 200 mM). Therefore, the results of the present study are in agreement and demonstrate that esterase activity can useful as a bioindicator of salinity in tomato plants. Studies related to β-galactosidase and NaCl-stress in tomato were not found in the literature; however, Daldoul et al. [88] reported a novel alkaline α-galactosidase (*Vv-α-gal-SIP*) in a *Vitis vinifera* salt-tolerant cultivar (Razegui) that was expressed under salt stress. A previous report was published on spinach salt tolerant cultivar where salt and drought stress stimulate the α-galactosidase expression in this cultivar [89]. Also, in *Vigna unguiculata* (L.) Walp. cultivars varying in water and salt stress tolerance, were isolated from cotyledons three isoforms of β-galactosidase; however, this study did not relate the isoforms to stress tolerance [90]. In rice, Lee et al. [91] showed that alkaline α-galactosidase is implicated in the hydrolysis for the glycolipid digalactosyldiacylglycerol (DGDG) which have been defined in the framework of abiotic stress response mainly water deficit in *Arabidopsis* [92] and *Vitis vinifera* [93]. Also, has been related DGDG as messenger in the pathway as a response to salt stress [90,94]. Different response showed the enzymes α-galactosidase, β-galactosidase, α-glucosidase and acid phosphatase in *Arachis hypogaea* L. under NaCl-stress (50 and 200 mM) which decreased as NaCl increased [95], which was attributed to a decreased wall elasticity.

## 4. Materials and Methods

### 4.1. Study Area

Two experiments were developed from May to July 2016 in La Paz, located in a semiarid zone of Baja California Sur, northwest of Mexico (24°08′ 09.73” N, 110°25′ 41.73′′ W), 7 m.a.s.l. Mean, maximum and minimum temperatures in the shade-enclosure were 29.0, 40.0 and 14.0 °C with 60% relative humidity during the tomatoes early vegetative growth stage (May to July). The climatic data were obtained from a computerized weather station located at the study area (Vantage Pro2 Davis Instruments^®^, Davis, CA, USA). The study site has a Bw (h’) hw (e) climate and is considered as a semiarid environment that sustains xerophytic flora [96].

### 4.2. Ethics Statements

The study area is not characterized as a protected area. The investigation developed herein did not include measurements with animals or humans. For locations/activities, no specific permissions were required and the field studies did not involve endangered or protected species. However, to carry out research activities on the lands administered by Centro de Investigaciones Biológicas del Noroeste, S.C. (CIBNOR^®^), permission was granted by the manager of the experimental open-field and shade-enclosure areas at CIBNOR^®^. The seeds of the tomato commercial varieties used were obtained from a store of agricultural products at La Paz, Baja California Sur, México. The species *Solanum lycopersicum* L. used in this study is not considered an in danger of extinction species and their use consequently had insignificant effects on wider ecosystem performance.

### 4.3. First Experiment

One independent experiment was carried out to determine the effect of NaCl on proline content and peroxidase specific activity of eight tomato commercial varieties.

#### 4.3.1. Plant Material and Experimental Conditions

Eight tomato commercial varieties, Yaqui, Missouri, Rio Grande (Saladette type) Ace, Tropic, Vita, Floradade and Feroz (Ball type) were evaluated for biochemical traits in the early vegetative growth stage. Seeds of separately tomato variety were sown into peat moss medium (May 1). The seeds were cultured under shade-enclosure conditions (30 °C day/20 °C night) during roughly 20 days under natural photoperiods (lat. 24°08′ 06.46′′ N). Plantlets were fertilized every 5 days with a Hoagland’s nutrient solution [97] and watered as needed. On May 22, plants were transplanted (plantlets with approximately 4 or 5 leaves and 10–15 cm of height) into plastic pots with a 25 cm height and 20 cm wide on the upper surface with about 4 kg of capacity and with holes at the bottom for drainage, containing a mixture of peat-moss (Sunshine, Sun Gro Horticulture^®^ Canada, Ltd., Santa María CA, USA) and sand and (1:1, *v*:*v*). The pots were arranged in a shade-enclosure and cultured under normal solar radiation. After being transplanted, 1 L of tap water was applied every 2 days for 10 days until plant rooting was developed. Homogeneous plants were chosen to be treated with NaCl. The shade-enclosure consisted in the use of a model of mesh 1610 PME CR, this meant 16 × 10 threads cm^−2^ with holes of 0.4 × 0.8 mm, and crystal color with 40% of shadow (monofilament stabilized polyethylene). The saline treatments were 0, 50, 100, 150 and 200 mM of NaCl (0.3, 2.6, 5.1, 7.6 and 10.3 dS m^−1^, respectively), which were applied after 10 days of tap water irrigation. All plants were watered daily with an excess of NaCl treatments mixed with one solution with concentrate nutrients (stock solution) containing (g in 3 L^−1^ of distilled water): 168 of KNO_3_, 30.6 of (NH_4_) (NO_3_), 44.4 of (NH_4_) H_2_PO_4_, 180.6 of Ca (NO_3_)_2_, 126 of MgSO_4_, 6.0 of FeSO_4_, 1.5 of MnSO_4_, 0.3 of ZnSO_4_, 0.3 of CuSO_4_ and 0.3 of H_3_BO_3_ in agreement with the suggestion of Samperio-Ruiz [98] for tomatoes. The second week began with the gradual implementation of the saline treatments, in order to avoid an osmotic shock in seedlings, according to the methodology of Murillo-Amador et al. [99]. The NaCl treatments occurred for 35 days after the transplanting process. The pH of all treatment solutions was maintained at close to 6.0 by adding H_2_SO_4_ or KOH. The plants were adequately fertilized, irrigated and protected from pests and diseases throughout the growing period according to local typical organic cultivation processes. Plants were grown with a 12-h photoperiod on photosynthetically active radiation (PAR) of 573.50 (max), 220.66 (min) and 393 ± 65 (average) µmol m^−2^ s^−1^.

#### 4.3.2. Proline Content

Free proline content was estimated following the method of Bates et al. [100]. Fresh 0.5 g of healthy leaves, uniform in color and size were collected from each plant and respective variety, NaCl and replication and then homogenized in 5 mL of 3% sulphosalicylic acid using a mortar and pestle. The, 2 mL of extract was taken in a test tube and to it 2 mL of glacial acetic acid and 2 mL of ninhydrin reagent were added. The reaction mixture was boiled in a water bath at 100 °C for 30 min. After cooling the reaction mixture, 4 mL of toluene was added. After thorough mixing, the chromosphere containing toluene was separated and the absorbance of red color developed was read at 520 nm against toluene black on a UV-visible spectrophotometer. The proline concentration was determined using a calibration curve and expressed as mg proline per g fresh weight of tissue.

#### 4.3.3. Peroxidase Specific Activity

The specific activity of peroxidase was determined by the Pütter and Becker [101] method. Previously, 10 g of fresh healthy leaves, uniform in color and size were collected from each plant, variety, NaCl and replication, respectively. All samples were stored in individual vials which were placed in an ultra-freezer at −70 °C. The samples were lyophilized (Virtis Benchtop ES^®^, Stone Ridge, NY, USA) at −51 °C with a vacuum less than 80 μBars for 20 h. Thirty milligrams of each lyophilized samples were taken and 3 mL of potassium phosphate buffer was added, before being homogenized (Virtis Benchtop ES^®^), allowed to stand for 1 h and centrifuged at 3600 rpm, 5 °C for 5 min (Eppendorf 5810R^®^, Hamburg, Germany). The supernatant was recovered and placed on ice until use. Quartz cells of 1 cm path length, 1.59 mL (final volume) were used to read the absorbance at 436 nm at 25 °C using a spectrophotometer (Beckman Coulter DU 800^®^, Brea, CA, USA).

#### 4.3.4. Experimental Design

A factorial experiment of two-ways of classification were used, with the eight tomato varieties previously mentioned as the first factor and five NaCl concentrations cited previously as the second factor, arranged in a completely randomized design with four replicates and each replicate consisting of one pot with three plants per pot.

#### 4.3.5. Statistical Analysis

Bartlett’s test was executed on the data to test the homogeneity of variance. Data were analyzed using multivariate-univariate analysis of variance (MANOVA and ANOVA) according to a factorial design of two-ways of classification, with tomato varieties (Missouri, Rio Grande, Vita Yaqui, Feroz, Ace, Tropic, and Floradade) and NaCl concentrations (0, 50, 100, 150 and 200 mM de NaCl) modeled as fixed factors, with their interactions for a completely randomized design. Distilled water was used as control (0 mM NaCl). The differences between the means were determined by Tukey’s HSD multiple range test at *p* = 0.05. The data were analyzed using Statistica^®^ v. 13.5, Palo Alto, CA, USA [102].

### 4.4. Second Experiment

In this independent experiment, the enzymatic activity profiles through semi-quantitative enzymes determination was developed, using germinated seeds of the eight tomato commercial varieties with root and shoot that grew for 14 days. The experiment was conducted under laboratory conditions.

#### 4.4.1. Germination Test

Seeds were disinfected by immersion for 5 min in calcium hypochlorite solution, containing 1% chlorine. The seeds were then washed two times with sterilized distilled water. Afterwards, the seeds were deposited in sterilized Petri dishes ((150 × 15 mm) covered at the bottom with a filter paper (Whatman^®^ No. 1, Darmstadt, Germany)). Each Petri dish was soaked with 5 mL of the proper NaCl solution (0, 50 or 100 mM of NaCl). Petri dishes were sited in a germination chamber (Lumistell^®^, model IES-OS, series 1408-88-01, Celaya GTO, MX, USA) at 25 °C ± 0.5 °C with 80% of relative humidity. Fourteen days old seedlings were divided into shoots and roots for semi-quantitative enzyme determination.

#### 4.4.2. Experimental Design

The experiment was arranged according a factorial experiment of two-ways of classification, with tomato varieties (Yaqui, Missouri, Feroz, Vita, Rio Grande, Tropic, Ace, and Floradade) as a first factor and three NaCl concentrations (0, 50 and 100 mM de NaCl) as a second factor, in a completely randomized design with four replicates. Distilled water was used as control (0 mM NaCl). The tomato seeds of all varieties were subjected to each NaCl concentrations for 14 days.

#### 4.4.3. Semi-Quantitative Enzymes

The study of semi-quantitative enzymatic activity in tomato subjected to different NaCl concentrations was determined by the API ZYM^®^ system (bioMérieux^®^ Inc., Hazelwood, MI, USA). The API ZYM^®^ consists of a plastic gallery of cupules, in the bottom of each of which is a fabric provision carrying the substrates and buffer. The API ZYM^®^ contain of 20 microcupules with dehydrated chromogenic substrates of 19 enzymes and a control (a microcupule that does not contain any enzyme substrate). These enzymes include eight glycosyl-hydrolases (β-galactosidase, β-glucosidase, N-acetyl-β-glucosaminidase, α-glucosidase, α-galactosidase, β-glucuronidase, α-mannosidase and α-fucosidase), three phosphatases (alkaline phosphatase, acid phosphatase and phosphohydrolase), three aminopeptidases (leucineamino-peptidase, valine amino-peptidase and cystine amino-peptidase), three esterases (lipase, esterase-lipase and esterase) and two proteases (chymotrypsin and trypsin). One gram of fresh shoots and roots of each variety was obtained and crushed using aseptic distilled water (1:100) under room laboratory temperature (20 °C). This solution was filtered to remove impurities, shaken for 5 min, allowed to settle for 5 min and the supernatant of fresh fluid was used for enzyme analysis with the following procedure: an amount of 65 µL of the fresh fluid was dispensed to all strip cupules containing dehydrated substrates for enzymes. After incubation at 25° C for 24 h in the dark, the galleries were activated by adding one drop of ZYM A^®^ reagent (Tris-hydroxymethylaminomethane, hydrochloric acid 37%, sodium lauryl sulfate, H_2_O) and one drop of ZYM B^®^ (Fast Blue BB, 2-methoxyethanol). After 5 min at room temperature, semi-quantitative evaluation of the activities was measured referring to a colorimetric standard table [103] by designating a numerical value of 0–5 (equivalent to 0–40 nmol) depending on the chromogenic substrate intensity produced by the hydrolase reaction. For the objectives of this study, the scores were registered as the percentage of the greatest enzymatic activity (API ZYM^®^) that might be attained. In order to determine the reproducibility of results using this technique, all of the varieties were examined using four replications, both for shoots and for roots.

#### 4.4.4. Statistical Analysis

From the 19 enzymes of API ZYM^®^ and before analysis of semi-quantitative enzyme activity, the enzymes β-Glucuronidase, α-Glucosidase, β-Glucosidase and α-Fucosidase for shoots and roots as well as Lipase (C14) for roots, were eliminated from the dataset; this was because they do not register any activity in any variety. Also, Yaqui, Feroz and Vita in both shoots and roots were not included in the statistical analysis because no enzyme activity was registered in any NaCl concentration. Later, canonical discriminant analysis was used to evaluate the intensity of the relationship between enzymes (API ZYM^®^) and the different tomato varieties were subjected to three NaCl concentrations (0, 50 and 100 mM NaCl). The procedure of canonical discriminant was used to remove variables within the model that do not offer extra information or were unnecessary as determined by the Wilks’ Lambda method, as well as to add variables outside the model that contributed best to the model [104]. Also, canonical discriminant was applied to create a discriminant function useful for determining the accuracy of the set of tomato varieties groups, based on the pooled covariance matrix and the prior probabilities of the classification groups. The data obtained were used to separate classification variables (tomato varieties) based on linear combinations of the dependent variables. The linear combinations of variables (canonical roots) were later correlated with the original groups. Canonical roots mean (centroid values) was calculated for each classification variable and the significance between means was determined using Mahalanobis distance. Individual values for each canonical root were plotted in a bi-plot for the first and second canonical roots. The original data (without discrimination analysis) of API ZYM^®^ activities of shoots and roots (average of four replicates samples per each NaCl concentration) were subjected to cluster analysis using the Euclidean distance measure. Dendrograms were constructed with the Ward Method. The Ward algorithm was applied to the similarity matrix generated from the intensity of the different activities (from 0 to 40 nmol) by using the Euclidean distance coefficient. Furthermore, the two-ways joining clustering method was used to show the intensity of color in the figures which is proportional to enzyme activity. All analyses were done with Statistica^®^ v. 13.5 [102] using modules multivariate exploratory techniques, as well as cluster and discriminant analysis.

## 5. Conclusions

Proline activity exhibited a differential activity among varieties and increased as NaCl increased, while peroxidase activity did not reveal differences among varieties and did not show a trend to decrease or increase under NaCl-stress. Acid and alkali phosphatase, lipase, esterase, β-galactosidase, and trypsin can be potential biomarkers for NaCl-stress tolerance in the seedlings stage of the tomato. Even though this study did not discuss the implications of increments of these enzymes in plants under NaCl-stress, this research could support a reference point and a method that are very important for conducting future studies in relation to the biochemical basis of NaCl-stress tolerance in tomato. According the proline content, the varieties with NaCl-stress tolerance were in the order of Vita > Floradade > Rio Grande > Yaqui > Feroz > Tropic > Missouri > Ace. This disparity response can be attributed to the systematic capacity of each variety to tolerate NaCl-stress or because of the effect of NaCl-stress could cause some change in gene expression. In general, the enzymatic activity and some minerals (K, Ca, Mg and others) decreases with increasing NaCl concentration; as a result, all genotypes that increase enzymatic activity under NaCl-stress can maintain their nutritional value, since some enzymes such as acid phosphatase can maintain a certain level of minerals in plant cells, while the enzymatic activity status is important to determine the nutritional value of fruits and vegetables. The advantage of APY-ZYM^®^ test was verified as a rapid and easy technique to determine semi-quantitative enzymatic activity in tomato and their relationship with NaCl-stress tolerance. This study is not conclusive in estimating that enzymes may have special metabolic functions during germination and seedling stage in tomato under NaCl-stress. Consequently, the functions of glycosyl-hydrolases, phosphatases, esterases and proteases under NaCl-stress continue to be theoretical and require more research.

## Figures and Tables

**Table 1:** Average data of proline and peroxidase activity including the interaction of both factors, with tomato varieties as the first factor and NaCl concentrations as the second factor.

	**Proline (mg g^−1^)**				
	**mM NaCl**				
**Varieties**	**0**	**50**	**100**	**150**	**200**
Missouri	2.16 ± 1.01 ^e*^	4.21 ± 1.44 ^c^	4.26 ± 1.36 ^b^	11.99 ± 2.74 ^b^	34.81 ± 0.79 ^a,b^
Ace	5.72 ± 1.48 ^d,e^	1.61 ± 0.19 ^c^	8.88 ± 2.95 ^b^	7.06 ± 0.50 ^b^	12.81 ± 4.90 ^c^
Yaqui	12.70 ± 1.79 ^c,d,e^	31.69 ± 2.98 ^a,b^	35.13 ± 0.61 ^a^	26.35 ± 5.56 ^a^	33.77 ± 1.11 ^a,b^
Feroz	24.37 ± 2.27 ^a,b^	9.4 ± 43.01 ^c^	34.77 ± 1.31 ^a^	30.79 ± 2.13 ^a^	18.64 ± 6.04 ^b,c^
Tropic	1.36 ± 0.33 ^e^	6.04 ± 0.85 ^c^	6.89 ± 1.47 ^b^	33.96 ± 1.57 ^a^	28.70 ± 3.76 ^a,b,c^
Rio Grande	15.09 ± 1.20 ^b,c,d^	23.67 ± 1.83 ^b^	31.05 ± 2.44 ^a^	34.13 ± 0.94 ^a^	31.60 ± 3.74 ^a,b^
Floradade	30.73 ± 2.09 ^a^	34.82 ± 2.01 ^a^	35.40 ± 0.46 ^a^	33.09 ± 1.54 ^a^	24.80 ± 2.09 ^a,b,c^
Vita	18.12 ± 5.48 ^b,c^	33.80 ± 1.35 ^a^	34.41 ± 1.35 ^a^	35.17 ± 0.94 ^a^	34.94 ± 0.57 ^a^
Mean	13.78	18.16	23.85	26.57	27.51
	**Peroxidase (µ mg of Protein min^−1^)**				
	**mM NaCl**				
	**0**	**50**	**100**	**150**	**200**
Missouri	0.11 ± 0.02 ^a*^	0.22 ± 0.03 ^a^	0.24 ± 0.10 ^a^	0.14 ± 0.08 ^a^	0.14 ± 0.02 ^a^
Ace	0.12 ± 0.02 ^a^	0.27 ± 0.06 ^a^	0.41 ± 0.13 ^a^	0.14 ± 0.05 ^a^	0.23 ± 0.11 ^a^
Yaqui	0.54 ± 0.16 ^a^	0.31 ± 0.18 ^a^	0.30 ± 0.11 ^a^	0.22 ± 0.14 ^a^	0.59 ± 0.43 ^a^
Feroz	0.23 ± 0.09 ^a^	0.12 ± 0.05 ^a^	0.34 ± 0.10 ^a^	0.34 ± 0.05 ^a^	0.28 ± 0.07 ^a^
Tropic	0.16 ± 0.03 ^a^	0.20 ± 0.02 ^a^	0.21 ± 0.03 ^a^	0.35 ± 0.22 ^a^	0.13 ± 0.05 ^a^
Rio Grande	0.41 ± 0.15 ^a^	0.18 ± 0.09 ^a^	0.25 ± 0.07 ^a^	0.22 ± 0.06 ^a^	0.28 ± 0.05 ^a^
Floradade	0.14 ± 0.05 ^a^	0.20 ± 0.05 ^a^	0.36 ± 0.17 ^a^	0.46 ± 0.14 ^a^	0.27 ± 0.11 ^a^
Vita	0.12 ± 0.03 ^a^	0.28 ± 0.09 ^a^	0.27 ± 0.10 ^a^	0.30 ± 0.10 ^a^	0.22 ± 0.09 ^a^
Mean	0.23	0.22	0.30	0.27	0.27

* The values are means ± standard error of four replications. Values within the same column with same letters are not significantly different (Tukey´s HSD multiple range test *p* ≤ 0.05).
